# Emerging infectious diseases and the depopulation of French Polynesia in the 19th century.

**DOI:** 10.3201/eid0204.960416

**Published:** 1996

**Authors:** P M Martin, C Combes


9. Johnson DW, Pieniazek NJ, Griffin DW, Misener L, Rose JB. Development of a PCR protocol for sensitive detection of *Cryptosporidium* oocysts in water specimens. Appl Env Microbiol 1995;61:3849-55.

### Emerging Infectious Diseases and the Depopulation of French Polynesia in the 19th Century

**To the Editor:** The same dynamics now considered factors in the emergence of infectious diseases may have been involved in the dramatic depopulation of French Polynesia in the 19th century. Temporal and geographic variation in the frequency and severity of infectious diseases are the result of the encounter and interaction of a population of parasites and a population of hosts. J. Musser reviewed the "bacterial side of the equation" (1). On the host side, there are two historical models that describe the influence of parasitism on human populations (2-4): 1) the South American model, in which new pathogens were introduced into native populations by the European conquistadores, causing the death of 50 million people; and 2) the African model, in which infectious diseases present in native populations protected them from the effects of colonization until modern times when the discovery of quinine and other efficient antipathogenic drugs provided added protection. The second model is well illustrated by the attempted colonization of Madagascar, where the French lost five men to war and 5,000 to malaria (2). This letter intends to illustrate the first model. We suggest that during their first contacts with European navigators in the very late 18th century and the 19th century, Polynesian islanders, much like populations in the South American model, were decimated by newly introduced infectious diseases.

It is difficult to know precisely which infectious diseases were present in Tahiti and the other French Polynesian islands before the arrival of the first Europeans. However, a study of Polynesian languages indicates that Bancroftian filariasis and leprosy were already present, while syphilis and other venereal diseases, influenza, and tuberculosis (TB) were probably unknown. Epidemic diarrhea and dysenteriae could have existed, although first reports mentioned that the oldest Polynesians "never heard of dysenteriae before" (5). In the Marquesian language, names exist for leprosy, bronchitis, abscesses, and impetigo.

The number of inhabitants in Tahiti, as well as in the Marquesas and the Austral Archipelago, was at first only estimated by European explorers. However, a precise census was performed as soon as missionaries and French authorities noted the high death rates in most of the islands (5,7,15,16). Tahiti was annexed by France in 1843; the first census was performed in 1848, and the population size was assessed approximately every 5 years until 1911.

Four major epidemic diseases (TB, typhoid, influenza, and smallpox) devastated the Marquesas from 1791 to 1863/64; approximately 80% of the population died. During that period, exchange of populations between the Marquesas Islands also increased, as a consequence of colonization. Thus, leprosy increased dramatically during the second half of the 19th century, to a prevalence of 4.11% in 1884 (6).

In Rapa, the remote, southern island of the Austral Archipelago, at least three epidemics were reported, resulting in the loss of more than 90% of the population. Although the cause of the first epidemic remained unknown, dysenteriae and smallpox were identified as causes of the second and third epidemics, respectively.

From Rapa, a missionary went to Mangareva in 1831 or 1832, and his visit there was followed by an epidemic that the natives attributed "to his god." He had to flee back to Rapa. The second recorded epidemic disease was "Chinese scabies" in 1865, which decimated the child population. Then, the warship "La Zélée" brought an epidemic of influenza in 1908. In 1910, TB and leprosy were reported "to spread rapidly" (7), and in 1911, the ship "La Gauloise" brought whooping cough to Mangareva.

In Tahiti and the Society Islands, the number and diversity of international and interisland exchanges, involving numerous commercial ships and whalers, make the origin of epidemics more difficult to trace.

However, at least five were reported successively in the Leeward Islands in 1843, 1848, 1854, and 1864 (7), and at least 11 in Tahiti: influenza (1772 to 1774), pulmonary TB (1775), dysentery following the passage of the ship of Vancouver (1790), dysentery after the passage of the whaler "Britania" (1807), disastrous influenza in 1820, whooping cough in 1840, smallpox in 1841, dysentery again in 1843, scarlet fever in 1847, measles in 1852-1854 (800 deaths were recorded) and typhoid fever after the passage of "La Magicienne" in 1877 (8).

Almost without exception, authors attributed the dramatic depopulation of French Polynesia during the 19th century to infectious diseases. Other causes, such as alcohol, opium, local wars, infanticides, and even orgiastic behavior were also mentioned as possible causes. Depopulation occurred to a similar extent in other South Pacific countries (9), e.g., the Cook Islands, Hawaii, Tonga, Samoa, and particularly Fiji, where 50% of the population died. Thus, after limited initial contact with persons exposed to infectious diseases, most of the Polynesian populations died. Why did it happen? Why were epidemics so intense and so severe? It is unlikely that clones of bacteria, viruses, fungi, or parasites with particularly high virulence were introduced into native populations since the long crossing by sailing boats would have selected clones with lower virulence. Moreover, epidemics are also intense and severe in animal populations when new infectious agents are introduced. In Hawaii, the introduction of *Plasmodium* from birds had catastrophic consequences for the local fauna (10).

Host population factors that may influence the spread of an infectious agent (i.e., the intensity of an epidemic) are diverse: 1) social disruption was certainly a major cause for the increase of leprosy and TB in the Marquesas during the 19th century: pacification of the archipelago by Dupetit-Thouars changed traditional behavior and destroyed tribal barriers against leprosy by permitting the development of interisland exchanges, thus contributing to the spread of both leprosy (within the Marquesas) and TB (from Tahiti to one Marquesas island, then between the Marquesas) (11); 2) the absence of most infectious diseases in Polynesia before the 18th century probably slowed the selection of behavioral methods of prevention and the development of traditional medicine; 3) a small population without exposure to infectious diseases would not have selected resistance genes against nonexistent infectious agents; and 4) the lack of population immunity probably had a major role in the spread of new infectious agents.

Host population factors that can influence the virulence of parasites (i.e., the severity of an epidemic) are less frequent. Successive epidemics of closely related viruses or bacteria can enhance the severity of the disease, as in dengue fever (12), or can inversely provide cross-protection, as was suggested between yaws and syphilis (13), whose causative organisms are almost indistinguishable. Reduced genetic polymorphism of 19th century Polynesians who had no immunity to infectious diseases could have contributed to the severity of epidemics in the South Pacific, as it was speculated for South America (4,14).

**Paul M. V. Martin* and Claude Combes†**
*Institut Territorial de Recherches Médicales Louis Malardé, Papeete, French Polynesia; and †Centre de Biologies et d'Ecologie Tropicale et Méditerranéenne, Université de Perpignan, Perpignan, France

### References

1. Musser JM. Molecular population genetic analysis of emerged bacterial pathogens: selected insights. Emerging Infectious Diseases 1996;2:1-17.
2. Combes C. Interactions durables: écologie et évolution du parasitisme. Paris: Masson, 1995.
3. Haldane JBS. Disease and evolution. Ricerca Scientifica Supplement 1949;19:68-76.
4. Black FL. Why did they die? Science 1992;258:1739-40.
5. De Bovis E. Etat de la société tahitienne à l'arrivée des européens. Papeete: Société des Etudes Océaniennes, 1978.
6. Clavel CL. Les Marquisiens. Arch Med Nav 1884;42:194-212.
7. Toullelan PY. Tahiti colonial. Paris: Publication de la Sorbonne, 1987.
8. Vigneron E. Hommes et santé en Polynésie Française. Montpellier, 1991.
9. Mac-Arthur N. Islands population of the Pacific. Canberra: Australian National University Press, 1968.

10. van Ripper III C, Van Ripper SG, Lee Goff M, Laird M. The epizootiology and ecological significance of malaria in Hawaiian land birds. Ecological Monographs 1986;56:127-44.
11. Buisson GPE. Les îles Marquises et les Marquisiens. Annales d'Hygiène et de Médecine Coloniales 1903;6:535-59.
12. Halstead SB. Pathogenesis of Dengue: challenge to molecular biology. Science 1988;239:476-81.
13. Turner TB. Studies on the relationship between yaws and syphilis. American Journal of Hygiene 1937;25:477-506.
14. Garenne M, Aaby P. J Infect Dis 1990;161:1088.
15. Rollin L. Moeurs et coutumes des anciens Maoris des îles Marquises. Papeete: Stepolde, 1974.
16. Hanson A. Rapa, une île polynésienne hier et aujourd'hui. Paris: Publications de la Société des Océanistes, 1973.
17. Vallaux F. Mangareva et les Gambier. Papeete: Etablissement Territorial d'Achats Groupés, 1994.

### Epidemic Zoster and AIDS

**To the Editor:** Zoster (exogenously reactivated varicella-zoster virus infection) may seem an unlikely candidate for emergence and epidemicity. A recent report, however, describes a zoster outbreak associated with epidemic HIV in injecting drug users in Manipur State, India (1). In addition to underscoring the variety of ways in which "old" diseases may reemerge under complex bio-ecologic conditions, this outbreak may also have implications for anticipating and diagnosing HIV infections and AIDS in developing countries. The Manipur outbreak was associated with a doubling of zoster frequency above background levels, with increased occurrence most notable in males 12-44 years old, who also had the highest HIV prevalence. In a separately studied group of 120 injecting drug users, 20 developed zoster and all were found to be HIV positive (1), a correlation substantially greater than for such other clinical predicters of HIV infection as persistent lymphadenopathy, weight loss, or recurrent dermatoses. Increased zoster occurrence associated with HIV transmission has also been seen in Ho Chi Minh City, Vietnam, and in other Southeast Asian countries, particularly in injecting drug using populations (unpublished). Zoster as a sentinel indicator of community HIV transmission is also suggested by reports from Africa (2).

For over 150 years, it was believed that zoster occurred in local epidemics (3,4). By the 1950s, however, it was generally agreed that zoster represented reactivation of latent gangliar varicella virus either sporadically, or in response to immunosuppression or trauma. Epidemics of "endogenous" immunosuppression, such as those associated with epidemic HIV infection, might thus be expected to produce outbreaks of zoster, as seems to have occurred in Manipur and Vietnam. In the Indian outbreak traumatic zoster seemed unlikely: truncal and facial dermatomes predominated, rather than dermatomes corresponding to drug injection sites (usually the hands or legs). Recognition of zoster outbreaks may be important in developing countries where HIV diagnosis is limited, CD4 cell counts are unavailable, and diagnosis of AIDS is delayed. Zoster is not currently accepted as an AIDS-defining condition (5), and the extent to which it may reflect immune collapse or predict HIV disease progression is uncertain. Nevertheless, greater awareness of zoster as a sentinel indicator of community HIV transmission may be of help not only in clinical diagnosis, but also in public health efforts to recognize epidemic HIV occurrence.

**D. M. Morens,* A.K. Agarwal,† S. Sarkar,† S. Panda,† and R. Detels‡**
*University of Hawaii School of Medicine, Honolulu, Hawaii; ICMR Unit for Research on AIDS in North-eastern States of India, Calcutta, India†; and University of California at Los Angeles, Los Angeles, California‡

### References

1. Panda S, Sarkar S, Mandal BK, Singh TBK, Lokendra Singh K, Mitra DK, et al. Epidemic of herpes zoster following HIV epidemic in Manipur, India. J Infect 1994;28:167-73.
2. Tyndall MW, Nasio J, Agoki E, Malisa W, Ronald AR, Ndinya-Achola JO, Plummer A. Herpes zoster as the initial presentation of human immunodeficiency virus type 1 infection in Kenya. Clin Infect Dis 1995;21:1035-7.
3. Simon L. *Questions sur diverses branches des sciences médicales. Thèse 202.* Paris: Rignoux, 1840.
4. Kaposi M. Bemerkungen über die jüngste Zoster-Epidemie und zur Aetiologie des Zoster. Wien Med Wochenschr 1889;25:961-4, 26:1001-4.

